# The Influence of Oxidative Stress on Neurological Outcomes in Spontaneous Intracerebral Hemorrhage

**DOI:** 10.3390/biom11111615

**Published:** 2021-11-01

**Authors:** Julia Masomi-Bornwasser, Elena Kurz, Christina Frenz, Jan Schmitt, Dominik M. A. Wesp, Jochem König, Johannes Lotz, Florian Ringel, Thomas Kerz, Harald Krenzlin, Naureen Keric

**Affiliations:** 1 Department of Neurosurgery, University Medical Center of the Johannes Gutenberg University, 55131 Mainz, Germany; elena.kurz@unimedizin-mainz.de (E.K.); christinafrenz@web.de (C.F.); schmitt.j93@gmx.de (J.S.); dominik.wesp@unimedizin-mainz.de (D.M.A.W.); florian.ringel@unimedizin-mainz.de (F.R.); thomas.kerz@unimedizin-mainz.de (T.K.); Harald.Krenzlin@unimedizin-mainz.de (H.K.); naureen.keric@unimedizin-mainz.de (N.K.); 2 Institute of Medical Biostatistics, Epidemiology and Informatics (IMBEI), University Medical Center of the Johannes Gutenberg University, 55131 Mainz, Germany; koenigjo@uni-mainz.de; 3 Institute of Clinical and Laboratory Medicine, University Medical Center of the Johannes Gutenberg University, 55131 Mainz, Germany; Johannes.Lotz@unimedizin-mainz.de

**Keywords:** oxidative stress, intracerebral hemorrhage, neurological outcome, oxidative stress markers, cerebrospinal fluid

## Abstract

Spontaneous intracerebral hemorrhage (ICH) causes, besides the primary brain injury, a secondary brain injury (SBI), which is induced, amongst other things, by oxidative stress (OS) and inflammation, determining the patient’s outcome. This study aims to assess the impact of OS in plasma and cerebrospinal fluid (CSF) on clinical outcomes in patients with ICH. A total of 19 ICH (volume > 30 cc) patients and 29 control patients were included. From day one until seven, blood and CSF samples were obtained, and ICH volume was calculated. OS markers, like malondialdehyde (MDA), superoxide dismutase (SOD), glutathione peroxidase (GSH-Px), glutathione-sulfhydryl (GSH), and the total antioxidant status (TAS) were measured. Clinical data on treatment and outcome were determined. Patients with mRS ≤ 4 showed significantly elevated SOD and GSH-Px levels in plasma compared to patients with poor CO (*p* = 0.004; *p* = 0.002). Initial increased TAS in plasma and increased MDA in CSF were linked to an unfavorable outcome after six months (*p* = 0.06, r = 0.45; *p* = 0.05, r = 0.44). A higher ICH volume was associated with a worse outcome at week six (*p* = 0.04, r = 0.47). OS plays a significant role in SBI. Larger ICHs, elevated MDA in CSF, and TAS in plasma were associated with a detrimental outcome, whereas higher plasma-SOD and -GSH-Px were associated with a favorable outcome.

## 1. Introduction

Non-traumatic intracerebral hemorrhage (ICH) accounts for two million of all strokes (10–15%), with an incidence of 10–30 per 100,000 population per year worldwide [1]. Due to a high mortality rate of about 60% after one year and severe morbidity, this devastating stroke subgroup poses a significant public health burden [1,2]. Despite an increasing number of studies, controversy persists concerning the benefit of surgical evacuation compared to conservative treatment of ICH [3]. Large clinical trials, such as the Surgical Trial in Intracerebral Hemorrhage (STICH I and STICH II) or the Minimally Invasive Surgery Plus Recombinant Tissue-Type Plasminogen Activator for Intracerebral Hemorrhage Evacuation (MISTIE), failed to prove the clear advantage of either surgical strategies such as evacuation via a craniotomy hemicraniectomy or less invasive, catheter-directed lysis therapy by fibrinolytic drugs on long-term functional outcome [3,4,5,6,7,8,9,10].

Hemorrhage-induced primary tissue disruption and secondary progression due to destructive processes within the perihematomal zone cause neuronal injury after ICH. In addition, it is thought that the breakdown of hemoglobin to iron ions, heme, and thrombin induces the production of free radicals leading to direct neuronal damage [11].

Further, inflammation in the wake of ICH leads to the accumulation of large amounts of reactive oxygen species (ROS), resulting in oxidative stress (OS), consecutive neurotoxicity, disruption of the blood–brain barrier, and consecutive vasogenic edema [3,11]. ROS usually subsumes free radicals (e.g., superoxide anion, hydroxyl radical, peroxyl, and alkoxyl radicals) and species that can generate radicals in situ (hydrogen peroxide, singlet oxygen, hypohalous acids). Oxidative stress occurs due to an imbalance between antioxidant defense mechanisms and ROS [11,12,13]. Once initiated, it induces the damage of membrane lipids and DNA and the impairment of cellular protein function [14].

Defense mechanisms include antioxidant enzymes that eliminate superoxide anion radicals such as superoxide dismutase (SOD) and glutathione-sulfhydryl peroxidase (GSH-Px), which catalyzes the elimination of organic hydroxy peroxides [11,15,16]. Because ROS have an exceptionally short half-life, they are exceedingly difficult to detect [17]. Various biomarkers such as lipid peroxides, peroxidation products of DNA, and proteins have been described as measuring OS indirectly [11]. Malondialdehyde (MDA), a byproduct of lipid peroxidation, has well been established as an OS marker [11,18,19]. However, measurements of the serum total antioxidant status (TAS) as the overall cumulative measure became common practice instead of individual antioxidants [11,20]. Markers of increased OS are also detectable in cerebrospinal fluid (CSF) [21,22]. However, little is known about the interrelation of OS in plasma and CSF in patients with ICH [22].

This study analyzed markers of OS in plasma and CSF after ICH and its importance for neurological outcome.

## 2. Materials and Methods

### 2.1. Patients

The study was conducted in the University Medical Center of the Johannes Gutenberg University, Mainz, Germany. From January 2017 to January 2019, nineteen consecutive patients (7 female (37%) and 12 male (63%)) presenting on admission with an ICH of more than 30 mL, necessitating external ventricular drainage (EVD) (RAUMEDIC^®^ NEUROVENT, Helmbrechts, Germany), were included in our analysis. Further inclusion criteria were age ≥ 18 years; spontaneous, non-traumatic ICH, with or without the involvement of the ventricles; and diagnosed by CT imaging within 4 h of symptom onset in our department. Four of these patients (21%) additionally received hematoma evacuation via craniotomy. Twenty-nine patients without ICH served as controls (15 female (52%) and 14 male (48%)). The mean age of the ICH group was 64.05 ± 10.99 years, and 57.52 ± 15.28 years for the control group (Table 1). The inclusion criterion for the control cohort was CSF collection via a drain or craniotomy. Patients suffering from any kind of bleeding, infection, or inflammation were excluded.

### 2.2. Blood and CSF Samples

Five mL of EDTA-blood and four mL of CSF were obtained on days one (d1), three (d3), and seven (d7) after ICH. The CSF of the controls was obtained from either lumbar drainage placed for normal pressure hydrocephalus evaluation or the intraoperative opening of CSF spaces. All samples were centrifugated in 3000× *g* for ten minutes. The supernatant was aliquoted and stored at −80 °C until analysis.

### 2.3. Analysis of Antioxidants and Oxidative Stress Markers

SOD (Superoxide Dismutase Assay Kit (item no. 706002) from Cayman Chemical, Ann Arbor, MI, USA), GSH/ GSH-Px (the Ransel Glutathione Peroxidase Kit (cat. no. RS 504), Randox Laboratories Ltd. Crumlin, United Kingdom), TAS (ImAnOx (TAS/TAC) Kit (KC5200), Immundiagnost AG (64625 Bensheim, Germany), and MDA (HPLC-reagent kit (Item No. 66000 and 67000), Chromsystems Instruments & Chemicals GmbH (82166 Gräfelfing, Germany)) were analyzed in CSF and plasma respectively using colorimetry, high-performance liquid chromatography (HPLC) or ELISA.

### 2.4. Evaluation of Clinical Data and Imaging

CT scans performed on the day of admission and at day seven were used for semiautomatic volumetric analysis by Sectra^®^ software (IDS7, Sectra Medical, Linköping, Sweden).

On admission, the Glasgow Coma Scale (GCS) and the National Institutes of Health Stroke Scale (NIHSS) were compiled. The outcome was evaluated using the modified Rankin scale (mRS) the Glasgow Outcome Scale Extended (GOSE) at six weeks and six months after ICH.

### 2.5. Statistical Analysis

All data are shown as means ± SD. We used the parametric T-test relations between SOD, GSH-Px, GSH, TAS, and MDA for statistical analysis. In addition, clinical outcome parameters were analyzed by one-way analysis of variance (ANOVA) with Bonferroni’s correction as post hoc analysis. Statistical Package for the Social Sciences (SPSS; version 23.0) software was used for statistical analyses. Correlations have been evaluated by Pearson correlation. The *p*-value < 0.05 indicated statistical significance.

### 2.6. Ethics Declarations

The Ethics Committee of the Medical Council of Rhineland Palatinate Germany approved this study (837.374.16) following the guidelines for clinical studies conducted at the University Medical Centre of the Johannes Gutenberg University Mainz. Data analysis and collection were performed anonymously. Informed consent was obtained before inclusion in this study.

## 3. Results

### 3.1. Baseline Demographics and Follow-Up

The median GCS on admission was 7 ± 4.94, and the median NIHSS was 11.5 ± 7.41 points. Twelve patients had follow-up visits after six weeks and six months, while seven were lost to follow-up. Median historic mRS was 1 ± 1.66. The mRS deteriorated to 5 ± 1.28 six weeks after ICH and remained at 5 ± 1.34 after six months. GOSE was 2.5 ± 1 at week six and improved to 3 ± 1.58 six months after ICH. The number of male and female patients was comparable (*p* = 0.322) as well as the age of the ICH and control patients (*p* = 0.115).

### 3.2. Superoxide Dismutase (SOD) in Plasma and CSF

**Figure 1:**
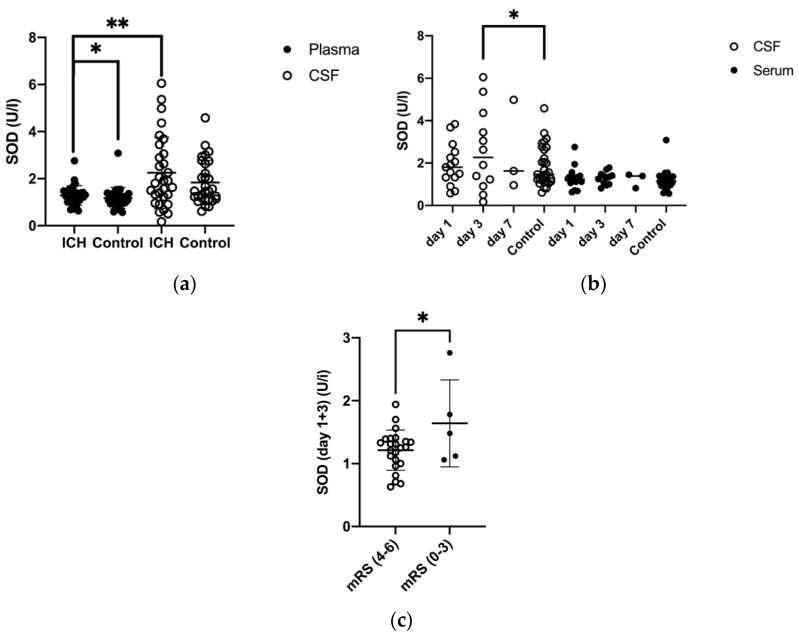
(**a**) Comparison of mean SOD levels in plasma (P) and cerebrospinal fluid (CSF) in ICH patients and control group, SOD (P) in ICH and control: *p* = 0.023, mean SOD (P) and mean SOD (CSF): *p* = 0.008 (**b**) Development of SOD levels in P and CSF in ICH patients and control group (day 1, day 3, and day 7), SOD (CSF) at day 3 and control: *p* = 0.02 (**c**) Comparison of SOD in P (day 1 and 3) of patients with a favorable (0–3) and an unfavorable (4–6) mRS after 6 months (*p* = 0.04), *p* ≤ 0.05 *, *p* < 0.01 **, *p* < 0.001.

The mean SOD concentration in plasma (of days one, three, and seven) showed a significantly higher value in ICH patients than in the control patients (1.51 ± 0.8 U/I vs. 0.45 ± 0.49 U/I, *p* = 0.03). Nonetheless, it was significantly lower than mean CSF levels in ICH patients (*p* = 0.008) (Figure 1a). The mean SOD concentration in CSF of ICH patients was elevated but not significantly compared to the control patients (2.41 ± 1.65 U/I vs. 0.94 ± 0.94 U/I). There was a slight increase in the SOD levels at different time points in CSF (Figure 1b) (Table 2).

In contrast, SOD levels in CSF increased at day three to significantly higher than the baseline of controls (*p* = 0.02) (Figure 1b).

ICH patients’ plasma level of SOD (day one and three) with a worse clinical outcome after six months (defined as mRS > 4) was 1.21 ± 0.32 U/L, which was significantly lower than the SOD level of patients with a favorable clinical outcome of 1.64 ± 0.69 U/L defined as mRS ≤ 4 (*p* = 0.004) (Figure 1c).

### 3.3. Glutathione Peroxidase (GSH-Px) in Plasma and CSF

**Figure 2:**
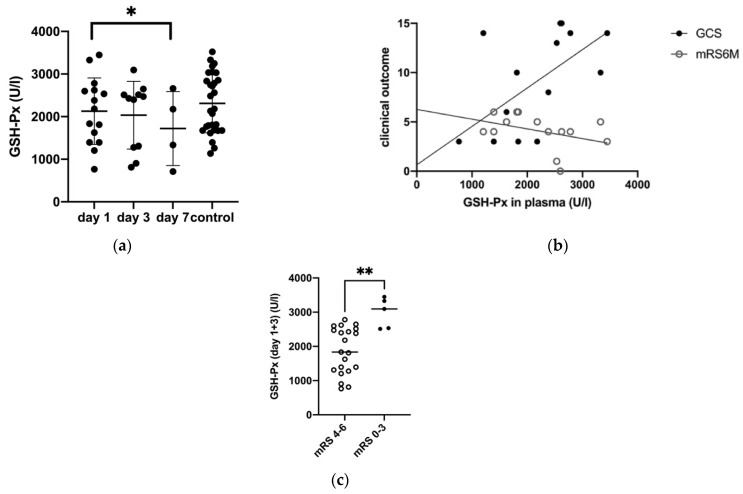
(**a**) Development of GSH-Px levels in plasma (P) in ICH patients and control group (day 1, day 3, and day 7), GSH-PX at day 1 and 7: *p* = 0.046 (**b**) Correlation of GSH-Px levels in plasma with mRS after 6 months (*p* = 0.04) and with GCS in ICH patients (*p* = 0.03) (**c**) Comparison of GSH-Px in P (day 1 and 3) of patients with a favorable (0–3) and an unfavorable (4–6) mRS after 6 months (*p* = 0.002), (*p* ≤ 0.05 *, *p* < 0.01 **).

From day one to seven, the level of the antioxidant enzyme glutathione peroxidase (GSH-Px) decreased significantly in ICH patients (day one: 2127.23 ± 201.52 U/L, day seven: 720.0 ± 433.88 U/L; *p* = 0.046) (Figure 2a, Table 2).

A low plasma GSH-Px level at day one was significantly correlated with a poor neurological status at the time of admission (GCS; *p* = 0.03, CI 95% = 6.86–139.42 U/L, r = 0.48) and an unfavorable outcome after 6 months (mRS) (*p* = 0.04, CI 95% = −0.002–0.00, r = 0.53) (Figure 2b). A plasma level of GSH-Px at day one and three after ICH in patients with an unfavorable outcome after six months (defined as mRS > 4) was 1874.4 ± 676.3 U/L and significantly lower than the GSH-Px in patients with a favorable clinical outcome 2983.4 ± 439.2 U/L (defined as mRS ≤ 4) (*p* = 0.002) (Figure 2c).

### 3.4. Glutathione-Sulfhydryl (GSH) in Plasma

**Figure 3:**
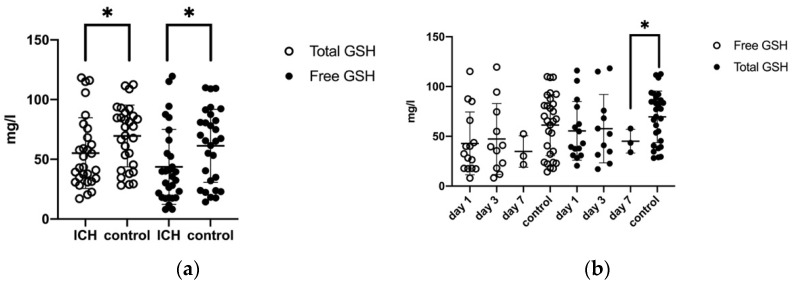
(**a**) Comparison of mean total GSH and free GSH in plasma (P) in ICH patients and control group, free GSH and control: *p* = 0.02, total GSH, and control: *p* = 0.03 (**b**) Development of free and total glutathione levels in plasma in ICH patients and the control group (day 1, day 3, and day 7), total GSH (P) at day 7 and control: *p* = 0.04, (*p* = ≤0.05 *).

The mean serum level of total GSH (at days one, three, and seven) was significantly reduced in the ICH patients (54.04 ± 28.7 mg/L) compared to the control patients (69.6 ± 25.8 mg/L; *p* = 0.03). Likewise, mean free GSH concentration in plasma was reduced after ICH (42.83 ± 30.4 mg/L vs. 61.3 ± 30.6 mg/L, *p* = 0.02) (Figure 3a). On days one, three, and seven the total and free GSH concentrations showed a tendency to decrease without reaching statistical significance (55.5 ± 7.59 mg/L, 57.65 ± 10.36 mg/L, 45.1 ± 6.9 mg/L and 42.86 ± 8.15, 47.33 ± 10.74, 34.78 ± 9.14) (Figure 3b; Table 2). Neither clinical conditions at the onset nor clinical outcomes over time correlated with GSH.

### 3.5. Total Antioxidant Status (TAS) in Plasma and CSF

**Figure 4:**
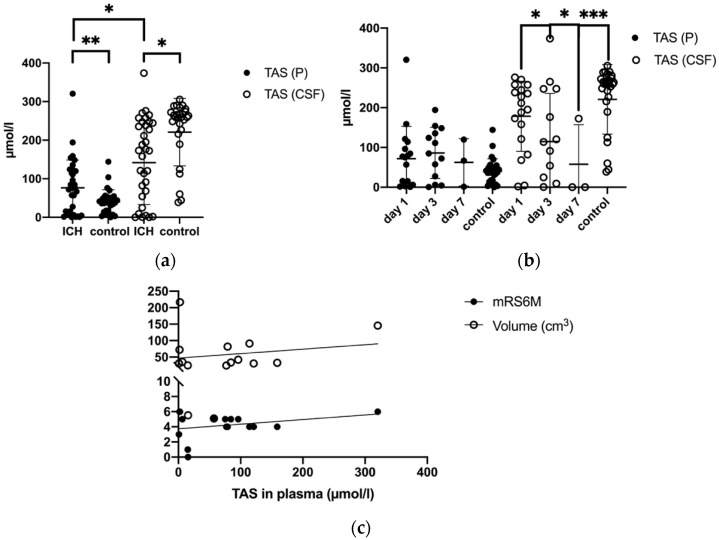
(**a**) Comparison of mean TAS levels in plasma (P) and cerebrospinal fluid (CSF) in ICH patients and control group (**b**) Development of TAS levels in P and CSF in ICH patients and control group (day 1, day 3 and day 7), TAS (CSF) day 1, day 3 and day 7: *p* = 0.03, *p* = 0.03, TAS (CSF) at day 7 and control: *p* = 0.0001 (**c**) Correlation of TAS levels in P to mRS after 6 months and to the volume of ICH in ICH patients, *p* = ≤0.05 *, (*p* = <0.01 **, *p* = <0.001 ***).

While the mean TAS was significantly higher in plasma in patients with ICH (82.41 ± 74.26 μmol/L) compared to controls (40.01 ± 31.4μmol/L, *p* = 0.002), it was markedly lower in CSF after ICH compared to controls (ICH: 135.28 ± 109.1 μmol/L vs. controls: 220.99 ± 87.4 µmol/L, *p* = 0.002) (Figure 4a, Table 2). Among ICH patients, mean TAS (day one, three, and seven) was significantly higher in CSF (135.28 ± 109.1 μmol/L) than in plasma (82.41 ± 74.26 μmol/L, *p* = 0.016). Plasma TAS levels at day one, three, and seven after ICH decreased from 80.25 ± 20.43 μmol/L at day one to 86.22 ± 17.82 µmol/L at day three and 62.60 ± 34.41 µmol/L at day seven. TAS levels in CSF showed a similar trend (Figure 4b, Table 2). Higher TAS in plasma at the time of ICH significantly correlated with an unfavorable clinical outcome (mRS) after six months (*p* = 0.06, CI 95% = 0.0–0.014, r = 0.45) and with an increasing volume of hemorrhage from day one till seven (*p* = 0.03, CI 95% = −0.75–−0.13, r = 0.48) (Figure 4c). However, this observation could not be confirmed in CSF.

### 3.6. Malondialdehyde (MDA) in Plasma and CSF

**Figure 5:**
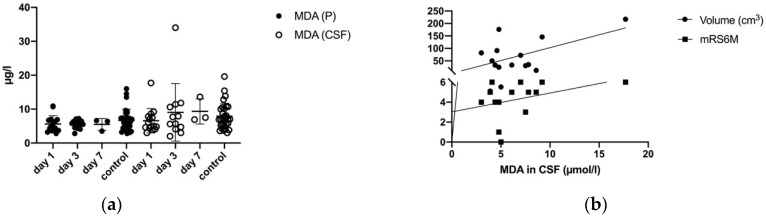
(**a**) Development of MDA levels in plasma (P) and CSF in ICH patients and control group (day 1, day 3, and day 7) (**b**) Correlation of mean MDA levels in CSF with mRS after 6 months (*p* = 0.05) and MDA (CSF) at day 1 with the volume of ICH (*p* = 0.01).

ICH had an impact on the mean MDA concentrations (days one, three, and seven) in plasma and CSF. From day one until seven, the MDA levels of the ICH and control patients remained comparable in plasma (day one: 5.63 ± 2.43 mg/L; day two: 5.44 ± 1.30 mg/L, day three: 5.50 ± 1.73 mg/L; control: 6.67 ± 3.33 mg/L) (Figure 5a, Table 2). The MDA level in CSF tended to increase at day one 6.57 ± 3.60 mg/L, three 9.04 ± 8.50 mg/L and seven 9.33 ± 3.71 mg/L compared to the control group 7.92 ± 3.83 (Figure 5a, Table 2).

Increasing MDA levels in CSF from day one until seven significantly correlated with an unfavorable clinical outcome after six months (mRS; *p* = 0.05, CI 95% = −0.002–0.38, r = 0.44).

In CSF, increased MDA levels showed a significant correlation with the ICH’s increased volumes (*p* = 0.01, r = 0.53, CI 95% = 1.5–192) (Figure 5b).

**Figure 6:**
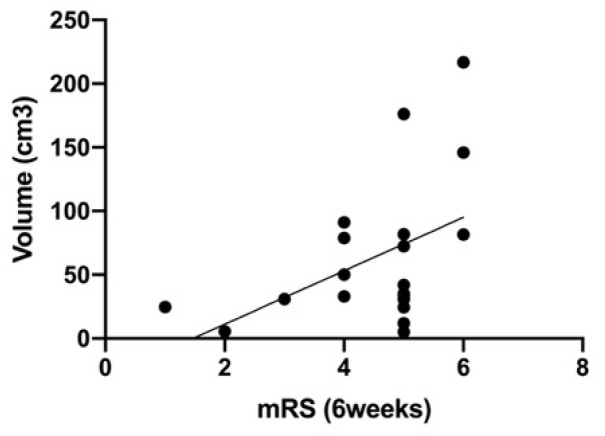
Correlation of mRS after 6 weeks and the volume of ICH at admission (*p* = 0.05).

Increased initial ICH volumes correlated significantly with a worse clinical outcome at week six (mRS; *p* = 0.04, CI 95% = 0.59–42.83, r = 0.47) (Figure 6).

## 4. Discussion

Our study showed that increased SOD and GSH-Px plasma levels after ICH are associated with a favorable neurological outcome after ICH. In contrast, elevated plasma TAS correlated with a poor outcome six months after hemorrhage. Furthermore, higher MDA levels were detected within the CSF in patients with larger ICH volumes, leading to an unfavorable outcome after six months.

Despite an increasing number of studies addressing ICH interventions, only marginal improvement of the clinical outcome has been achieved over the last decades [5,6,7,9,23,24,25,26]. A possible reason for this missing clinical therapy success could be that ICH has to be seen and treated as a multifactorial disease. A wide-scattered field of factors like size, location of the ICH, the primary brain injury, the patient’s health status and drugs, the secondary brain injury, the surgical volume-reducing therapy, the time until treatment, and blood degradation products and their toxicity and inflammation influence this complex disease [1,11,27]. The underlying pathophysiological mechanisms of secondary deterioration remain poorly understood. Recently, the role of free radicals and oxidative stress as a physiological response and in pathological processes after ICH have become the center of attention [28]. The majority of studies have failed to demonstrate clinical benefits by targeting OS. This might be due to a heterogeneous methodology, as measurement of oxidative stress remains error-prone. Reasons for this might be the high complexity of various reactions, the heterogeneity of ICH patients, sample collection, storage, methods, and time points of measurement. Furthermore, there is a persisting difficulty in deciding whether changes are the cause or result of oxidative stress. [11,15]. A target for drug development is desired but still distant from reality.

There are two significant pathways through which SBI and OS are interrelated [11,29]. Firstly, the activation of inflammation by OS and blood degradation products leads to a release of free radicals leading to excitatory amino acid production, destruction of ion transporters of the cell membrane, increased intracellular calcium, decreased ATP, activated phospholipase, disruption of organelles and cell membranes, acidosis, and finally cytotoxic edema. Secondly, free radicals stimulate the production of nitric oxide (NO), matrix metalloproteinases (MMPs), and other inflammatory cytokines and lead to a disruption of the blood–brain barrier and consecutive vascular edema [30]. Perihemoragic edema (PHE), which consists of cytotoxic and vasogenic edema, develops from the beginning until the subacute phase (after 72 h). It is the well-known and often described morphological consequence of complex inflammatory and molecular cascades [31]. PHE mainly consists of serum proteins from hematoma coagulation and, to a lesser degree, of CSF precipitation through the low resistance of the perivascular space. CSF plays an important and still not understood role in developing PHE and SBI, which strongly correlate with each other [32].

These changes cause damage to neuronal cells via a direct membrane and DNA damage and the initiation of apoptosis [11,33]. SOD and GSH-Px are antioxidative enzymes that protect against free radicals like superoxide anion radicals and organic hydroxy peroxides [11,15,34]. A significant increase in serum SOD and GSH-Px after ICH has been reported in previous studies. Although its upregulation protects against OS, the accumulation of both enzymes is associated with multi-organ damage and early brain injury in ischemic stroke and ICH [11,15,16]. After ICH, SOD levels decrease due to exhaustion [11].

Our data confirmed a considerable neuroprotective role of elevated SOD plasma levels in ICH patients. Plasma levels of SOD were higher in patients with a favorable outcome six months after hemorrhage than those with unfavorable outcomes. As SOD was not measured later after ICH, its potential consumption could not be observed. Our results align with previous data reporting lower concentrations of plasma SOD in patients with ICH [15]. The importance of SOD in ICH has been proven in a recent study on cell replacement therapy in mice. Here, neural stem cells (NSCs) overexpressing SOD1 led to increased neuronal survival and mitigated the detrimental effects of ICH [35]. In patients with ischemic stroke, SOD activity was lower and inversely related to infarction size [36]. So far, little is known about SOD in CSF after hemorrhagic or ischemic stroke. It is believed that SOD levels in CSF increase after ischemia and decrease after SAH [37]. In our study, SOD in CSF was higher in patients with ICH than controls without being statistically significant. Furthermore, CSF SOD levels did not influence the neurological outcome in our cohort. Thus, the importance of SOD in CSF after ICH remains a matter of debate.

There are contradictory results regarding the role and concentration of the intracellular antioxidant enzyme GSH-Px after ICH. GSH-Px is believed to play a neuroprotective role by reducing phospholipid hydroxide and lipid peroxidation [11,15,38]. In our study, early low GSH-Px plasma levels were associated with a poor clinical condition at the time of admission and an unfavorable outcome after six months. Over time, a statistically significant decrease in plasma GSH-Px levels after ICH was observed. Patients with a favorable clinical outcome had significantly higher mean GSH-Px levels than those with an unfavorable outcome.

GSH, a non-enzymatic antioxidant, is a cofactor of the GSH-Px family. GSH is an oligopeptide capable of reducing and inactivating oxidized proteins and pro-oxidant xenobiotic agents. Oxidized GSH is reduced by glutathione reductase [11,16].

A decline of cerebral GSH-levels was observed in association with cell death in animal ICH models. Treatment with exogenous GSH led to neuroprotection and amelioration of neuronal damage by the up-regulation of mitochondrial oxidative respiration function in a rat ICH model [39,40]. Here, we observed a significant decline of total and free GSH levels in the plasma of patients with ICH compared to those of controls. However, there was no correlation of GSH with the neurological outcome.

Plasma levels of MDA, a marker for lipid peroxidation caused by OS, are increased after ICH and correlated with mortality [11,18,19,41]. Although MDA levels did not differ significantly from controls in our study, an increase in MDA in CSF was associated with an unfavorable outcome six months after ICH.

In addition to measuring single antioxidants, the total antioxidant status was measured in plasma and CSF. Previous studies provided conflicting data regarding the TAS. It is believed that TAS is decreased in plasma after ICH [11,20]. In contrast, studies showed an ultra-early increase in TAS plasma levels in patients with sepsis, stroke, and traumatic brain injury. Higher early TAS levels are associated with higher mortality, potentially compensating for increased ROS levels in those patients [42,43,44]. In our cohort, TAS in CSF was significantly higher than in plasma but lower than in the CSF of the controls. The TAS in CSF decreased further until day seven after ICH. Elevated TAS levels in plasma were related to an increased ICH volume and an unfavorable clinical outcome after six months. As TAS decreased until day seven, a biphasic course constituted by reactive increase, followed by a reduction due to consumption, seems likely as in ischemic stroke patients [43]. This observation in our patients falls in line with studies showing a decreased TAS after ICH [11,20]. However, verification of this hypothesis would require a more extended observation period and a higher number of patients and measurements.

OS is becoming a potential target for drug development aiming to lessen brain damage after ICH. The two main strategies include increasing the TAS and lowering the level of ROS. Various studies evaluated natural, enzymatic antioxidants like SOD, GSH-Px, NADPH, catalase (CAT), peroxidase, and non-enzymatic antioxidants like vitamin C, E, GSH, copper, zinc, selenium, melatonin, and others. Other treatments pursue reducing the OS by hydrogen sulfide or targeting the OS-signaling pathway using Rosiglitazone. Some drugs relate to the statin or iron-chelating family or the scavenger for oxygen free radicals like Edavarone [11,19]. The role of these novel approaches as future complimentary ICH therapies remains a matter of ongoing research, but a targeted treatment for SBI is still not found.

There are clear limitations of our study. In general, there is a limited sample size of only 19 ICH and 29 control patients. Due to the small number of patients, correlations of OS biomarkers with the clinical outcome have to be considered with caution. They may not reflect the correlation observed in our cohort. Further clinical studies investigating blood and CSF biomarkers of ICH with a larger sample size and more extended observation periods are necessary to investigate the underlying mechanisms. We a priori excluded patients who received an intrathecal application of fibrinolytic or other drugs. Besides this, other factors potentially influencing OS, like systemically applied drugs or smoking, were not analyzed, representing a shortcoming of our study.

## 5. Conclusions

Our study shows that oxidative stress is involved in the pathomechanisms after ICH, impacting the clinical outcome. Increased SOD and GSH-Px plasma levels after ICH were associated with a favorable neurological outcome after ICH, while elevated plasma TAS correlated with a detrimental outcome six months after hemorrhage. Besides this, higher MDA levels were detected within the CSF in patients with larger ICH volumes, leading to an unfavorable outcome after six months. Future prospective studies, including ones with more patients and with a transparent symptomatic methodology, are necessary to determine the importance of ROS and oxidative stress for secondary injury development as a therapeutic target.

## Figures and Tables

**Table 1:** Baseline data.

Characteristics	ICH Group	Control Group
Number of patients	19	29
Sex, n [%]		
Male	12 (63%)	14 (48%)
Female	7 (37%)	15 (52%)
Age [years]		
Mean (SD)	64.05 ± 10.99	57.52 ± 15.28
Mean volume of hemorrhageat day 1 [cm^3^]	69.75 ± 57.4	-

**Table 2:** Antioxidative/Oxidative Parameters.

Antioxidative/ Oxidative Parameters	ICH Group			Control Group
	Day 1	Day 3	Day 7	Day 1
SOD (P)	[U/I]			
Mean (SD)	1.18 ± 0.13	1.29 ± 0.26	1.22 ± 0.20	0.45 ± 0.49
SOD (CSF)	[U/I]			
Mean (SD)	1.84 ± 0.25	2.58 ± 0.55	2.52 ± 1.67	0.94 ± 0.94
GSH-Px (P)	[U/I]			
Mean (SD)	2127.23 ± 201.52	2033.84 ± 239.38	1720.0 ± 433.88	2312.72 ± 695.89
Free GSH (P)	[mg/L]			
Mean (SD)	42.86 ± 8.15	47.33 ± 10.74	34.78 ± 9.14	61.34 ± 30.60
Total GSH (P)	[mg/L]			
Mean (SD)	55.5 ± 7.59	57.65 ± 10.36	45.1 ± 6.9	69.57 ± 25.82
TAS (P)	[μmol/L]			
Mean (SD)	80.25 ± 20.43	86.22 ± 17.82	62.60 ± 34.41	40.01 ± 31.4
TAS (CSF)	[μmol/L]			
Mean (SD)	178.40 ± 20.80	114.87 ± 31.25	57.67 ± 57.37	220.99 ± 87.4
MDA (P)	[mg/L]			
Mean (SD)	5.63 ± 2.43	5.44 ± 1.30	5.50 ± 1.73	6.67 ± 3.33
MDA (CSF)	[mg/L]			
Mean (SD)	6.57 ± 3.60	9.04 ± 8.50	9.33 ± 3.71	7.92 ± 3.83

## Data Availability

All datasets analyzed in this study are available with the corresponding author on reasonable request.
